# Who inhabits the built environment? A microbiological point of view on the principal bacteria colonizing our urban areas

**DOI:** 10.3389/fmicb.2024.1380953

**Published:** 2024-05-28

**Authors:** Jessica Zampolli, Alessandra De Giani, Massimiliano Rossi, Margherita Finazzi, Patrizia Di Gennaro

**Affiliations:** Department of Biotechnology and Biosciences, University of Milano-Bicocca, Milan, Italy

**Keywords:** microbiomes, microbial community, human health, human microbiota, environmental niches, omic approaches, urban cities

## Abstract

Modern lifestyle greatly influences human well-being. Indeed, nowadays people are centered in the cities and this trend is growing with the ever-increasing population. The main habitat for modern humans is defined as the built environment (BE). The modulation of life quality in the BE is primarily mediated by a biodiversity of microbes. They derive from different sources, such as soil, water, air, pets, and humans. Humans are the main source and vector of bacterial diversity in the BE leaving a characteristic microbial fingerprint on the surfaces and spaces. This review, focusing on articles published from the early 2000s, delves into bacterial populations present in indoor and outdoor urban environments, exploring the characteristics of primary bacterial niches in the BE and their native habitats. It elucidates bacterial interconnections within this context and among themselves, shedding light on pathways for adaptation and survival across diverse environmental conditions. Given the limitations of culture-based methods, emphasis is placed on culture-independent approaches, particularly high-throughput techniques to elucidate the genetic and *-omic* features of BE bacteria. By elucidating these microbiota profiles, the review aims to contribute to understanding the implications for human health and the assessment of urban environmental quality in modern cities.

## 1 Introduction

In recent decades, the world has witnessed a significant shift toward urbanization, with more than half of the global population now residing in cities. This trend is expected to continue, with projections indicating that by 2050, two-thirds of the world’s population will be urban dwellers (Stanley et al., 2023). Moreover, approximately 90% of people living in developed countries spend their lives principally indoors, moving from homes to workplaces by cars and public transport systems (Kelley and Gilbert, 2013; National Academies of Sciences, Engineering, and Medicine et al., 2017; Rai et al., 2021).

**Figure 1:**
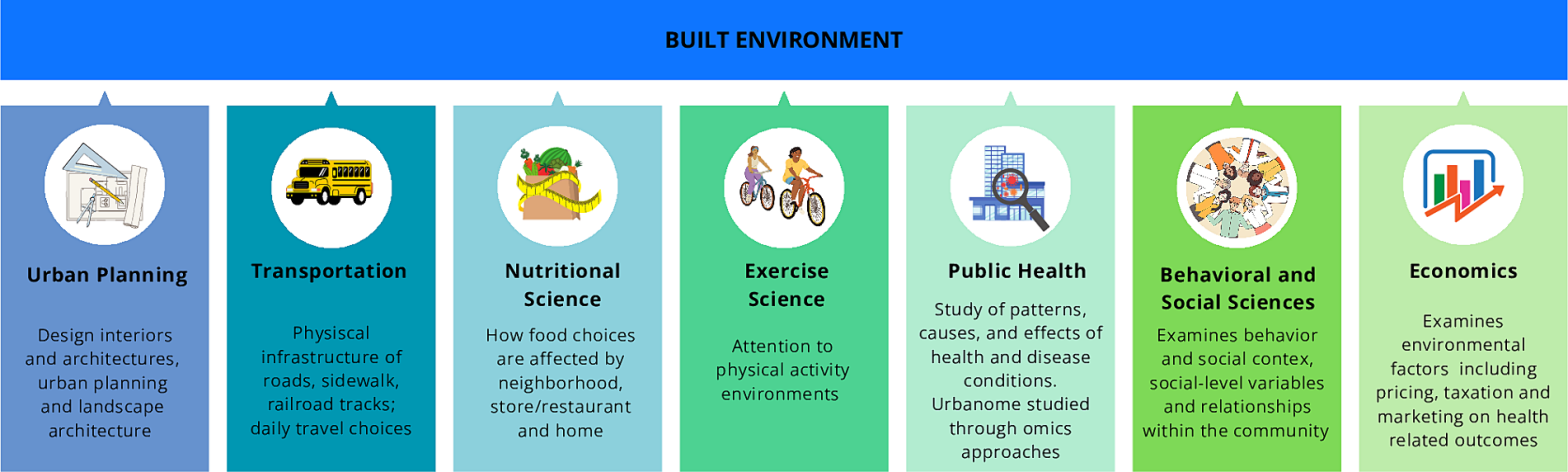
Main disciplines enable the evaluation of the built environment concept and quality and assess potential interventions for human health and well-being. The built environment assessment is based on different fields of study: urban planning, transportation, nutritional science which cooperates with behavioral (psychology), social, and environmental science, physical activity, epidemiology and public health, and economics.

The built environment (BE), encompassing the human-made spaces where individuals live, work, and socialize, plays a pivotal role in this urban landscape (Roof and Oleru, 2008; Gilbert and Stephens, 2018). In the early 2000s, BE was conceived as a framework for understanding the physical aspects of human habitation, the concept of BE has evolved to encompass broader considerations, including its impact on human health and well-being (Renalds et al., 2010; Glanz et al., 2016; Ciric, 2022) (Figure 1).

A BE can be considered as a new ecosystem on Earth, surely characterized by the presence of microorganisms in all its parts. The definition encompasses different living micro-entities, such as bacteria, fungi, protozoa, and algae. The occurrence of these microscopic inhabitants in our buildings became evident in the second half of the XX century when scientists began to associate quantitatively the presence of fungal spores in the air and allergy symptoms in people attending specific built environments (BEs). The first investigations were conducted with culture-dependent approaches. Then, advancements in molecular biology, such as ribosomal RNA sequencing, have enabled researchers to explore the microbial composition of BEs in greater detail (Gilbert and Stephens, 2018).

BE microorganisms are not autochthonous but derive from different sources (Gilbert and Stephens, 2018), such as soil, water, and air, but also pets, and humans (National Academies of Sciences, Engineering, and Medicine et al., 2017; Gilbert and Stephens, 2018). Indeed, humans are the principal vector and the main source of bacterial diversity in the BE (Kelley and Gilbert, 2013). According to the Human Microbiome Project, huge microbial sources are human oral and nasal cavity, vagina, intestine, and skin representing the five research priorities in the scientific community (Peng et al., 2022). The oral and the nasopharyngeal tracts could be considered important interfaces between man and the environment, and a certain microbial load can be transported through the aerosol. For instance, indoor air can carry around 10^4^–10^6^ bacteria per cubic meter (Hewitt et al., 2012; Gauzere et al., 2014; Gong et al., 2020). Likewise, the skin can release 1.5 million cells per hour carrying 15 million bacteria (Kelley and Gilbert, 2013). Consequently, human contact leaves a characteristic microbial fingerprint on surfaces and the surrounding environment, with notable representation from bacterial phyla such as Firmicutes (now Bacillota), Bacteroidetes (Bacteroidota), and Proteobacteria (Pseudomonadota), among others (Wilkins et al., 2016). However, the BE is immersed in the environment, which in turn shapes in a different way the human microbial fingerprint. Generally, urban buildings are placed on various types of soils, each influenced by varying degrees of urbanization. Research indicates that bacterial communities in urban green spaces resemble those in non-urban areas, with dominant phyla including α-and β-Proteobacteria, Actinobacteria (now Actinomycetota), Bacillota, Planctomycetes (currently Planctomycetota), and Bacteroidota (Brevik et al., 2020; Nugent and Allison, 2022). While shifts in bacterial composition between soil types may not be significant, microbial diversity within each phylum plays a crucial role in shaping the functions of the BE (Nugent and Allison, 2022). These microbial functions have implications for human health, particularly through the production of microbial volatile organic compounds (MVOCs). MVOCs can impact metabolic, immune, and endocrine processes by entering the body through inhalation or skin contact due to their unique chemical properties (Gilbert and Stephens, 2018).

However, the full comprehension of the complex and intricate relationships among various microbial species in diverse environments has not been fully examined. Therefore, it has become increasingly evident that the study of the BE microbial community’s composition as well as its functionalities is important to improve the quality of the environment and the health of its occupants. In this direction, in the last decades, a new form of thinking buildings emerged, especially about BE microbiome investigations and it is called “bio-informed design.” This concept relies on the idea of settling a healthy BE microbiome by constructing new edifices “by learning from nature’s best ideas” (Green, 2014; Selcuk and Avinc, 2021). This should be translated into the appropriate conditions for the successful colonization of microorganisms, such as humidity and moisture, nutrients, temperature, pH, and no inhibitory molecules (National Academies of Sciences, Engineering, and Medicine et al., 2017; Rai et al., 2021).

Despite all the studies regarding the composition of the different microbial communities present in each BE compartment and specific locations (for example, offices, subways, hospitals…), the definition of a “healthy” BE microbiota is still far (Dannemiller, 2019). Nowadays, it has been recognized that comprehension of bacteria composition and microbial diversity in close contact with BE inhabitants foster living more healthily. Thus, many urban regeneration projects are born around the industrialized world to try to achieve this important definition, which allows the integration of different knowledge, from microbiology and ecology to architecture. Indeed, from the beginning of the 21st century, the expression “urban regeneration” includes different aspects of the cities, leading to a renewal aimed at obtaining sustainable places (McDonald et al., 2009).

In this scenario, the present review aims to describe the bacterial populations in close contact with BE inhabitants, mainly students and workers, that stay for many hours per day indoors (i.e., in classrooms/university buildings, and offices) and use public transport. We focus on articles published between 2008 and 2023, as they provide insights into the microbiota of the buildings and their surrounding environments associated with human presence. In particular, we focus only on the bacterial population, excluding fungi and viruses of BE, since they play significant roles within the BE and are the main players of *omic* high-throughput studies on the relationship between indoor/outdoor environments with respect to fungi or other microorganisms. Due to sampling and data analysis challenges, research on BE viruses is limited (Prussin and Marr, 2015).

Our objective is to outline the microbiota profiles of urban environments, with a focus on their implications for human health. The review is organized into interconnected paragraphs. First, bacteria associated with urban areas and their interactions in the BE will be described differentiating between indoor and outdoor. Since culture-based methods for the study of the BE microbiota are not exhaustive, a specific focus on the culture-independent approaches based on high-throughput techniques will show how the genetic and *-omic* features could enhance our understanding of the bacterial world in the BE. Consequently, the phyla of the most represented bacteria in the BE will be presented together with their main features and native niches to frame the meaning of environmental quality and health of the human population of modern cities.

## 2 Bacteria associated with urban areas and their interactions in the BE: degradative capacities for human health

The bacteria considered in this review belong to different spaces of urban areas which represent the bacterial BE niches. However, the description of the niche of an organism is challenging, even if it is believed that related species share similar spaces due to their similar nutritional requirements (Hester et al., 2019). The definition is even more problematic in the case of BEs. Biodiversity inventories for microbes in urban systems are lacking compared to those for plants and animals, highlighting the need for further exploration (King, 2014). Unfortunately, microorganisms in the BE are often viewed as contaminants rather than valuable and beneficial organisms that interact with humans and building manufacturers (Horve et al., 2020). For this reason, as well as due to the complexity of the microbiota profiles of bacterial communities in the BE, a few points need to be addressed. (i) Evaluate the microbiological features characterizing differences of the urban BE especially between indoor and outdoor; (ii) understand how the bacteria interact with this context, and (iii) with each other.

### 2.1 The bacterial features in diverse BEs

**Figure 2:**
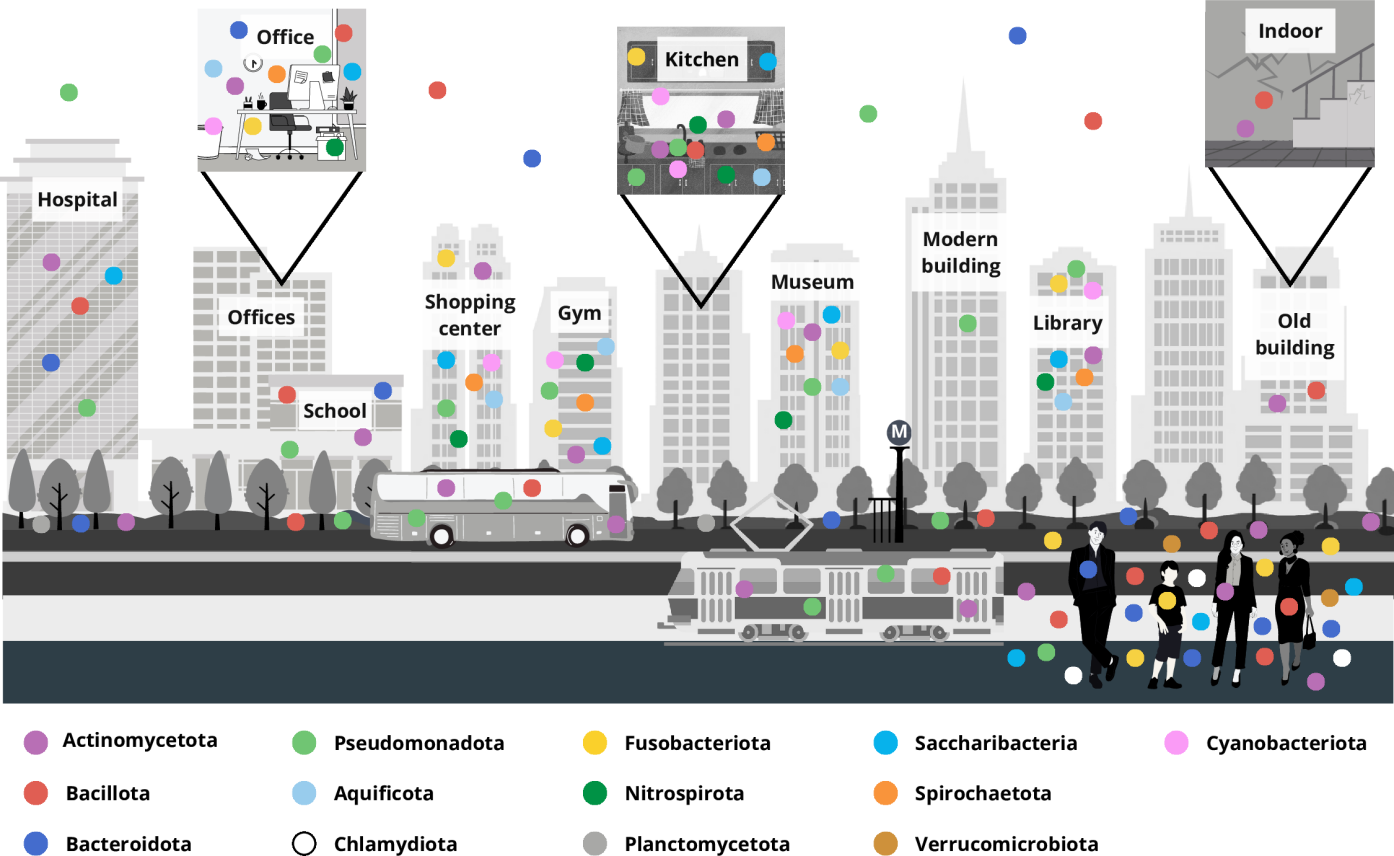
Representation of the microbiota distribution at the phylum level in a built environment localized in a typical modern city. The principal built environment places are marked, and square boxes represent different inner environments, for example offices, household kitchen and the indoor of an old building. Each colored dot represents a bacterial phylum, and their distribution approximates bacteria retrieved in the different compartments accordingly to the updated literature.

The bacteria of BE included in the considered research papers are principally grouped into four major phyla, Pseudomonadota, Bacillota, Actinomycetota, and Bacteroidota, and other less numerous phyla, namely Aquificae (now Aquificota), Chlamydiae (Chlamydiota), Fusobacteria (Fusobacteriota), Nitrospira (Nitrospirota), Planctomycetota, Spirochaetes (Spirochaetota), Saccharibacteria, and Verrucomicrobia (Verrucomicrobiota) along with Cyanobacteria (Cyanobacteriota) (Oren and Garrity, 2021) (Figure 2; Supplementary Table S1).

The Pseudomonadota phylum is highlighted for its prevalence in BE microbiota, and it is predominantly composed of Gram-negative bacteria, encompasses α-Proteobacteria, β-Proteobacteria, and γ-Proteobacteria (Supplementary Table S1). They are free-living non-parasitic, some of which are bacteria capable of fixing nitrogen and often comprise human-related bacteria and pathogens, and commonly found in various environments (Slonczewski et al., 2020). Among the six recognized classes within this phylum, 10 genera belong to the α-Proteobacteria and in the BE are found both outdoors and indoors in subways, offices, university classrooms, and associated with dust with a worldwide distribution (Kelley and Gilbert, 2013; Gauzere et al., 2014; Adams et al., 2015; Wilkins et al., 2016; Adams et al., 2017; Merino et al., 2019; Rai et al., 2021). Notable, genera like *Methylobacterium* and *Sphingomonas* are prevalent indoors, often originating from soil and water. Additionally, *Bradyrhizobium* (Hewitt et al., 2012; Kelley and Gilbert, 2013; Merino et al., 2019), *Neorhizobium* (Adams et al., 2017; Rai et al., 2021), and *Rhizobium* (Cao et al., 2021), typically associated with the rhizosphere, are also found in BE settings. Other members of α-Proteobacteria associated with humans such as *Bosea*, *Rhodobacter*, and *Brucella* are frequently detected indoors, including in offices, museums, and shopping centers (Wilkins et al., 2016; Gilbert and Stephens, 2018). In addition, several indoor spaces can host α-Proteobacteria; for instance, *Paracoccus* species are identified in the indoor bioaerosol of offices and museums (Gauzere et al., 2014; Adams et al., 2017), and *Brevundimonas* bacteria in the subway and the university classrooms (Adams et al., 2017; Merino et al., 2019).

Among β-Proteobacteria, *Bordetella*, *Burkholderia*, and *Neisseria* are prevalent indoors and associated with a high level of human occupancy (Kelley and Gilbert, 2013; Prussin and Marr, 2015; Gilbert and Stephens, 2018; Merino et al., 2019; Brevik et al., 2020; Rai et al., 2021). Some species of these genera can be pathogens that can be detected in offices, gyms, and indoor air with dust all over the world (Hewitt et al., 2012; Prussin and Marr, 2015; Gilbert and Stephens, 2018; Merino et al., 2019). Other genera, including *Delftia* and *Ralstonia*, are associated with the capacity to break down or transform a variety of pollutants, including plastic fibers (Merino et al., 2019; Peng et al., 2022).

Within reports about BE microbiota, 10 diverse genera belonging to γ-Proteobacteria are reported. The *Acinetobacter* and *Pseudomonas* genera are widely distributed in indoor BEs, including offices, and museums, with the first mostly in older buildings, while the second in modern ones. *Enterobacter* and *Escherichia* are also commonly found in various indoors as a sign of fecal contamination (Hewitt et al., 2012; Hospodsky et al., 2012; Kelley and Gilbert, 2013; Gauzere et al., 2014; Adams et al., 2015; Leung and Lee, 2016; Wilkins et al., 2016; Gilbert and Stephens, 2018; Merino et al., 2019; Cao et al., 2021; Rai et al., 2021; Leri and Khan, 2023). These genera also include pathogens such as *A. baumanii*, *A. johnsonii*, *P. aeruginosa*, *P. putida, Salmonella*, *Shigella*, *Legionella*, and *Vibrio harveyi*, posing health risks, since they have been detected in plumbing systems, water, cooling systems, and hospitals, emphasizing the importance of indoor microbial monitoring (Prussin and Marr, 2015; Browne et al., 2017; Gilbert and Stephens, 2018; Brevik et al., 2020; Horve et al., 2020; Rai et al., 2021).

The Bacillota phylum, dominated by Gram-positive bacteria, includes genera like *Bacillus* and *Staphylococcus*, prevalent in indoor environments such as offices, museums, and gyms worldwide (Hewitt et al., 2012; Kelley and Gilbert, 2013; Gauzere et al., 2014; Prussin and Marr, 2015; Leung and Lee, 2016; Gilbert and Stephens, 2018; Merino et al., 2019; Brevik et al., 2020; Horve et al., 2020; Egan et al., 2021). *Bacillus* species, metabolically adaptable and ubiquitously found, can be pathogenic and transmitted via ingestion, inhalation, or skin trauma such as *B. anthracis*, and *B. cereus* (Gilbert and Stephens, 2018); while *Staphylococcus*, commonly present on human skin and mucosal surfaces, is detected indoors and in the air of various buildings, such as mass transit railways, subways of big cities, university classrooms, ventilation duct supply, floor dust, and gyms (Hospodsky et al., 2012; Kelley and Gilbert, 2013; Adams et al., 2015; Prussin and Marr, 2015; Leung and Lee, 2016; Wilkins et al., 2016; Adams et al., 2017; Gilbert and Stephens, 2018; Merino et al., 2019; Rai et al., 2021; Panthee et al., 2022). Intriguingly, it has been detected in old buildings, due to a high level of human activities with respect to recently constructed edifices (Cao et al., 2021). A positive relationship between bacterial abundance and the urbanization level, human occupancy, and seasonal behavior in the BE has been noticed for *Streptococcus thermophilus* and *Enterococcus faecium* (Gilbert and Stephens, 2018). Other genera like *Paenibacillus*, known for their environmental presence, are occasionally detected indoors, specifically in the subway air (Merino et al., 2019). Additionally, lactic acid bacteria like *Lactobacillus* and *Lactococcus*, mainly associated with human presence, are found in indoor settings. Interestingly, due to the peculiar niche, the *Lactobacillus* genus can be associated with a female presence, while Corynebacteria as well as *Dermabacter* can be used as an index of male passage in the BE (Prussin and Marr, 2015). These last two taxa belong to the Actinomycetota phylum comprising Gram-positive bacteria with high G-C content (Gao and Gupta, 2012) which are widespread in the BE worldwide since they are associated both with the environment and human inhabitants. The bacteria of this phylum play diverse roles in the soil ecosystem, including organic matter degradation and antibiotic production (Servin et al., 2008; De Giani et al., 2021; Zampolli et al., 2022). Actinomycetota have also a beneficial role in humans, for instance *Bifidobacterium* genus in the GI tract (Presti et al., 2015; De Giani et al., 2022). Within this phylum, 10 genera are accounted in the BE, including *Corynebacterium*, *Mycobacterium*, *Propionibacterium*, *Streptomyces*, and *Rhodococcus* (Supplementary Table S1). *Corynebacterium* and *Mycobacterium* species are prevalent in offices, public transport systems, and museums, indicating human-associated microbial presence (Hospodsky et al., 2012; Kelley and Gilbert, 2013; Gauzere et al., 2014; Adams et al., 2015; Prussin and Marr, 2015; Leung and Lee, 2016; Wilkins et al., 2016; Gilbert and Stephens, 2018; Merino et al., 2019; Rai et al., 2021). Moreover, *Mycobacterium* subsp. can be opportunistic or the causative agents of nosocomial diseases. Interestingly, its presence and lifestyle can be also recognized in the BE by a few genomic traits (the relatively high GC content, the plasticity of the genome, the large genome size ranges, and the selected codon usage bias) (Gauzere et al., 2014; Adams et al., 2017; Browne et al., 2017; Gilbert and Stephens, 2018; Merino et al., 2019; Brevik et al., 2020; Horve et al., 2020). *Propionibacterium* and *Micrococcus* are prevalent in indoor bioaerosol worldwide (Gauzere et al., 2014; Prussin and Marr, 2015; Leung and Lee, 2016; Merino et al., 2019; Rai et al., 2021; Panthee et al., 2022). In particular, the *Propionibacterium* genus contains commensals of healthy human skin, nasopharynx, and oropharynx. Indeed, they are generally detected in humanized environments such as the air of mass transit railways and subways, settled-floor dust, and university classrooms (Hospodsky et al., 2012; Kelley and Gilbert, 2013; Adams et al., 2015; Prussin and Marr, 2015; Wilkins et al., 2016; Gilbert and Stephens, 2018; Merino et al., 2019; Rai et al., 2021). Other genera like *Actinomyces*, *Arthobacter*, *Rhodococcus*, and *Streptomyces*, primarily soil inhabitants (Hewitt et al., 2012; Wilkins et al., 2016; Zampolli et al., 2019; Cao et al., 2021; Zampolli et al., 2022), are occasionally detected indoors, reflecting their environmental presence.

Bacteroidota, encompassing Gram-negative bacteria distributed in almost all environments, human gut, and skin, includes genera like *Prevotella* and *Bacteroides* which are commonly detected indoors, particularly in household air and offices (Supplementary Table S1) (Hewitt et al., 2012; Prussin and Marr, 2015; Wilkins et al., 2016; Browne et al., 2017; Gilbert and Stephens, 2018; Merino et al., 2019). These genera, part of the human microbiota, play roles in processing dietary molecules and are indicative of fecal contamination, thus their levels increase with the urbanization level (Browne et al., 2017; Rai et al., 2021).

Additionally, other bacteria belonging to diverse phyla, Saccharibacteria, Fusobacteriota, Nitrospirota, Spirochaetota, Aquificota, and Cyanobacteriota are considered underrepresented in the BE, probably because they have shown low abundance levels, or due to the difficulty of taxonomic detection and assignment. They are detected mostly indoors, in offices, and in the bioaerosol reflecting origins and ecological roles (Griffiths and Gupta, 2006; Hewitt et al., 2012; Gauzere et al., 2014; Prussin and Marr, 2015; van Kessel et al., 2015; Horve et al., 2020; Oren and Garrity, 2021). These underrepresented taxa highlight the complexity of indoor microbial communities and their diversity.

### 2.2 Bacteria interactions in the BE

The BE is influenced by interrelated components such as physical, chemical, and (micro)biological factors, collectively known as the exposome. This encompasses exposure to external pollutants and internal host conditions (Dai et al., 2017). Indoor pollutants like carbon monoxide, heavy metals, volatile organic compounds (VOCs), and particulate matter (PM) accumulate on microplastic (MP) surfaces, forming a harmful mix that can accumulate and affect human health (Abdelsalam et al., 2020; Lindell et al., 2022; Nugrahapraja et al., 2022; Tamargo et al., 2022).

Bacteria can interact with xenobiotics in various ways, including biotransformation, growth inhibition, and bioaccumulation (Abdelsalam et al., 2020). For instance, a few reports show that xenobiotics found in the BE can be biotransformed by bacteria associated with human beings. Relevant pollutants for human health are phthalates, commonly found in plastics and clothing. They can be noncovalently bound to materials, thus they can be easily released into the environment and pose a risk for human ingestion, inhalation, or dermal absorption. If they reach different human districts, they can be modified into other chemicals (Chiu et al., 2020). Unfortunately, sometimes contaminants can be even biotransformed into more dangerous compounds (Abdelsalam et al., 2020). On the other hand, certain beneficial bacteria like lactobacilli can mitigate the toxicity of toxic compounds by enhancing the intestinal barrier (Feng et al., 2018). Despite the complexity of indoor pollutants, the literature emphasizes studying their combined effects rather than individual substances (Lindell et al., 2022). However, the study of the biotransformation of indoor pollutants remains underexplored due to the perceived inhospitality of indoor surfaces for bacterial growth, particularly in environments lacking sufficient nutrients and water (Hu and Hartmann, 2021). Computational studies reveal significant metabolic overlap in nitrogen and sulfur metabolism in the BE, suggesting unique adaptation strategies among bacteria inhabiting these spaces (Hester et al., 2019).

The consensus on microbial taxa abundance and frequency inside versus outside buildings varies in the literature. While some studies indicate distinct indoor and outdoor microbiota in terms of structure and composition (Rai et al., 2021), others suggest negligible differences in terms of the percentage of taxa evaluated by sequencing (Shin et al., 2015). For example, Actinomycetota, Pseudomonadota, and Bacillota percentages were comparable indoors and outdoors. Culture-based methods further support these findings, highlighting Gram-positive bacteria such as *Corynebacterium*, *Bacillus*, *Micrococcus*, and *Staphylococcus*, as predominant indoors, whereas among Gram-negative bacteria, *Chryseomonas* subsp. and *Pantoea* subsp. (Hewitt et al., 2012; Leung and Lee, 2016; Rai et al., 2021). Indoor environments consistently harbor higher bacterial concentrations in all seasons, particularly human-associated ones, evident in university classrooms and offices (Meadow et al., 2014; Adams et al., 2015). Microbes from outdoors (air, water bodies, soil, and vegetation) can infiltrate indoors through windows, ventilation systems, and passive transport via humans, pets, and plants. Human activities and outdoor microbial diffusion shape indoor microbial community structures (Rai et al., 2021). Additionally, potentially harmful bacteria may originate from anthropogenic sources like hospitals and wastewater treatment plants. For instance, *S. maltophilia* has exceptional metabolic persistence and versatility, thus it is often found in clinical settings, and *R. erythropolis*, known for its hydrophobic cells facilitating indoor colonization, is ubiquitously found (Leung and Lee, 2016; Cao et al., 2021; Rai et al., 2021).

Understanding microbial community assembly in BEs relies on deciphering microbe-microbe and microbe-environment interactions encoded in collective genome sequences. Despite abundant sequencing data, deciphering these sequences to understand community assembly and stability remains a challenge (Abreu and Taga, 2016). Co-occurrence patterns among bacterial taxa across communities can provide insights into functional roles, even for uncultured microorganisms (Barberan et al., 2012). Non-random co-occurrence suggests interactions shaping community assembly. Ecological network theory aids in identifying potential pathways for populating environments like new buildings. *In silico* analyses indicate that communities assemble tightly when species interact minimally, with strong host metabolic interactions limiting assembly (Coyte et al., 2021). Positive or mutualistic interactions lead to spatial co-occurrence, while competitive interactions result in co-exclusion, impacting population dynamics, species persistence, network stability, and ecological functions (Freilich et al., 2018). Integrating classical microbiology and *in silico* models can enhance understanding and guide bioinformed design in BEs, considering materials and usage patterns.

## 3 *Omic* approaches to detect and characterize the BE bacteria

**Figure 3:**
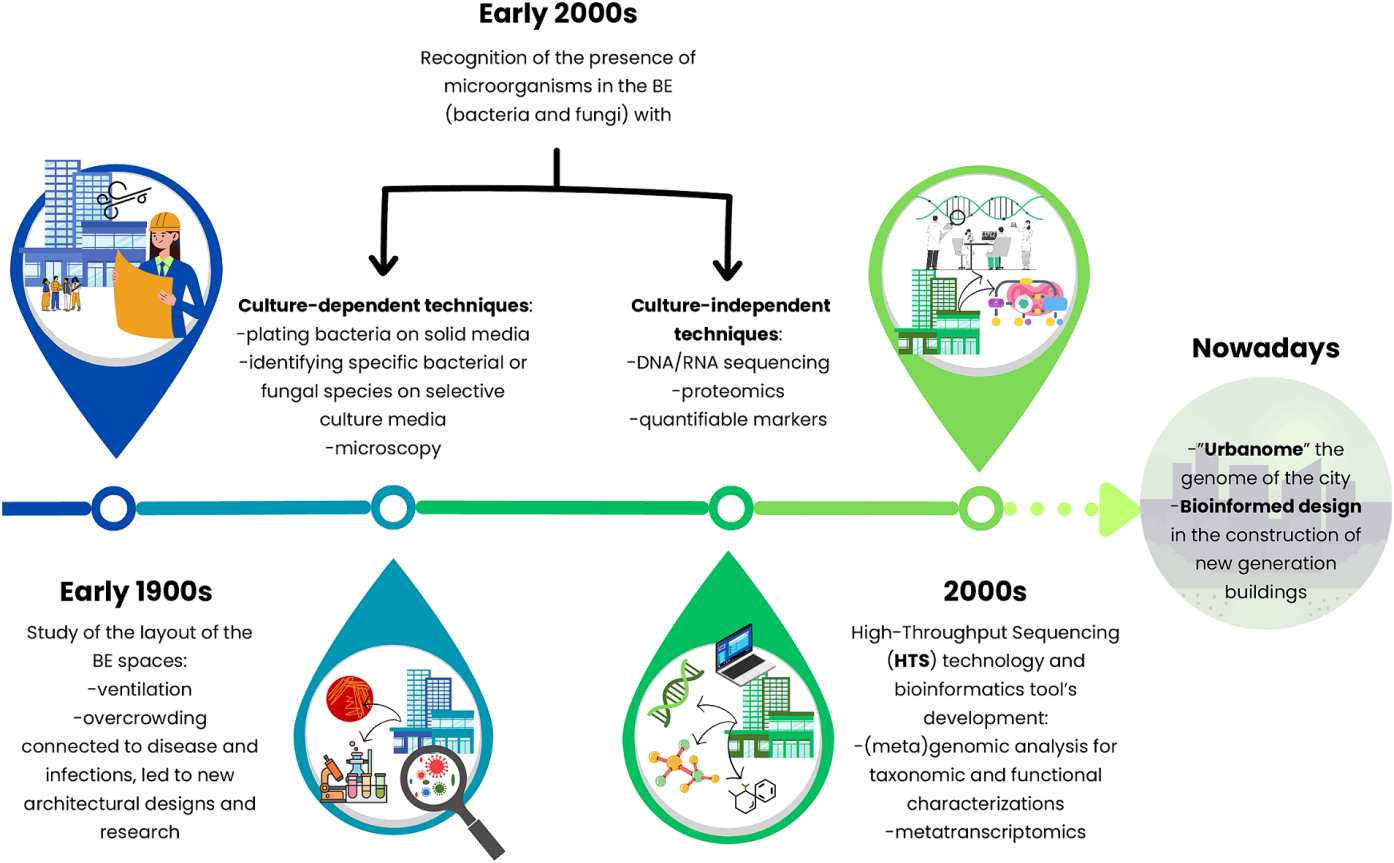
Timeline journey into the hidden properties of the microbiomes of the built environment at the global level from culture-based methods toward molecular-independent techniques and multidisciplinary approaches.

Since old times, ‘unclean’ indoor BEs have always been a concern for people, especially how they could negatively affect human health. Over the years, this concept has been linked to the presence of microorganisms living in different compartments of the BE, identified by culture-dependent techniques (Gilbert and Stephens, 2018). During the early 1900s, plating bacteria on solid media, identifying specific species on selective culture media, and counting microorganisms via microscopy were the first methods to study these anthropic systems. Later, researchers focused on tracking the sources, survival, and controlling microorganisms in the BE. However, not all microorganisms can be cultured with standard techniques (Daniel, 2004). Thus, the thriving of culture-independent approaches deepened the understanding of microbiology on previously unculturable microorganisms and the ecology of BEs (Figure 3).

A relevant issue raised by the literature is the microbial source characterization for the bacterial taxonomy investigation (Table 1). A widely used bioinformatics tool, called “source tracking” estimates the proportion of operational taxonomic units (OTUs) of a given community from potential “sources.” The sources could be outdoor air, soil, or humans, traceable by mRNA, proteins, or other quantifiable markers. Unfortunately, not all bacterial species can be associated with certain sources (Adams et al., 2015; Prussin and Marr, 2015).

**Table 1:** Selected research studies on BE microbiology using - omics approaches listed according to publishing release.

Year	-*Omic* technology	Target^a^	BE	References
2008	Metagenomics	Genomic DNA sequence and 6000 16S rDNA clones	Indoor microbiome of two shopping centers in Singapore	Tringe et al. (2008)
2012	Bar-coding with “universal” bacterial primers from 54 of the surfaces (18 per city) and pooled for pyrosequencing (culture-based cell counting and multiplexed pyrosequencing)	16S rRNA	30 different offices per city (90 offices, 450 total samples)	Hewitt et al. (2012)
2012	High-throughput sequencing	16S rRNA	Airborne bacterial community in patient rooms exposed to mechanical or window ventilation and in outdoor air	Kembel et al. (2012)
2013	Shotgun metagenomic sequencing	DNA	Indoor and outdoor air from diverse BEs in New York City and San Diego (USA; a large urban building, a medical center, a house, and a pier)	Yooseph et al. (2013)
2015	High-throughput sequencing (454, Illumina platforms)	16S rRNA	Indoor environment	Adams et al. (2015)
2018	Comparisons of methods and biological-based differences in both ribosomal transcript (rRNA) and gene (DNA) sequence community analysis	Pure strains and mock BE communities	Indoor air and surfaces	Gomez-Silvan et al. (2018)
2019	Metadata analysis (bacterial genera, BE location identified, sample type, temperature, humidity, and approximate climate)	Genomes (genome size, GC content, replication strand skew, and codon usage bias)	BE	Merino et al. (2019)
2019	Pangenomic meta-analysis (from GenBank)	189 genomes of two epidemiologically important taxa, *B. cereus* and *S. aureus*	International Space Station (ISS; a model BE), Earth-based BEs, soil, and humans	Blaustein et al. (2019)
2019	Metagenome-assembled genomes	Metabolic overlap connected to the functional redundancy of microbial communities at the genome scale	MO in diverse BE ecosystems	Hester et al. (2019)
2021	Illumina metagenome	Geospatial profile of microbial strains, functional characteristics, antimicrobial resistance (AMR) markers, and genetic elements	Mass-transit systems in 60 cities over 3 years	Danko et al. (2021)
2021	Illumina metagenome	Shotgun sequencing	Public transit air of 3 cities of 3 continents Denver, Hong Kong, London, New York City, Oslo, Stockholm	Leung et al. (2021)
2022	Illumina sequencing V4 e RT-qPCR	V4 16S rRNA and 18 kinds of ARGs	Indoor dust	Peng et al. (2022)
2022	-*Omic* platforms	Urbanome	Cities	Morawska et al. (2022)
2022	Multi-omics technologies (methylome, transcriptome, proteins and metabolites)	Human exposome	Multi-center cohort of 1,301 mother–child pairs to systematically associate a wide range of environmental exposures (>100 chemical, outdoor, social and lifestyle exposures) assessed in pregnancy and childhood	Maitre et al. (2022)
2023	Nanopore sequencing	16S-nanopore dataset generated by MegaBLAST, and culturable species based on the conventional culture results	46 urban samples from 18 stations of the mass transit system of Hong Kong in the period of July to October	Lee et al. (2023)
2023	Microbial DNA qPCR Array for ARGs; ddPCR; qPCR; NGS technologies	Culture-based (Rodac plates) and culture-independent methods (AMR, SARS-CoV-2, V3 region of 16S rRNA)	Milan subway	D’Accolti et al. (2023)
2023	Illumina sequencing	V4 region of 16S rRNA	Hand and built environments within the office and home settings	Hoisington et al. (2023)

a Target, each genetic and/or genomic quantifiable marker used for BE microbiome evaluations.

Since early 2000, High-Throughput Sequencing (HTS) technology and bioinformatic tools have had a rapid development with the project of human genome sequencing and the evaluation of the BE microbiome has been under the magnifying glass (Reuter et al., 2015). Diverse kinds of HTS were implemented over the years for the study of the different environmental compartments and the comprehension of BE inhabitants: (i) The 16S ribosomal RNA gene (rRNA gene) amplicon sequencing guarantees a taxonomic characterization mining the microbial diversity of a specific niche of BE (Hewitt et al., 2012; D’Accolti et al., 2023); (ii) (meta)genomic analysis enabled both the taxonomic and functional characterization of the community (Gilbert and Stephens, 2018); (iii) a deeper evaluation is additionally achieved by a metatranscriptomic approach revealing how certain conditions influence microbial populations. Consequently, a higher representation of transcripts signifies more expressed functions. However, to our knowledge, only one study applies metatranscriptomics for the BE (Hegarty et al., 2018) (Table 1).

The most applied approach is 16S rRNA amplicon sequencing and it has been correlated to, for example, the biodiversity of outdoor bacteria (from subways or mass transit stations), indoor airborne or office surfaces, or the abundance of antibiotic-resistant genes (ARGs) from dust microplastics (MPs) (Hewitt et al., 2012; Kembel et al., 2012; Peng et al., 2022; D’Accolti et al., 2023; Hoisington et al., 2023). One of the first studies exploited amplicon sequencing to study the microbiome of biofilm formed on vinyl shower curtains which harbored among other bacteria potential opportunistic pathogens (Kelley et al., 2004). Peng and coauthors (Peng et al., 2022) exploited the analysis of 16S rRNA V4 hypervariable region for exploring bacterial composition and biodiversity indoors, on dust and MPs. Moreover, the bacterial abundances were correlated to MP polymers and quantitative PCR of six main ARGs.

The prompt development of HTS led to the sequencing of full-length 16S rRNA by nanopore technology that implemented the taxonomy based on culture-based methods, particularly for the pathogenic species (Lee et al., 2023).

To overcome the mere taxonomy characterization of the BE microbiome, the whole genetic information began to be sequenced. In 2008, the first metagenomics study of the indoor microbiome was conducted by sampling air microorganisms of two shopping centers in Singapore. This study underlined that sampled microorganisms are not random transients from surrounding outdoor environments but originate indoors (Tringe et al., 2008). A shotgun metagenomic sequencing study conducted in urban areas of New York City and San Diego analyzed bacterial and fungal composition in indoor and outdoor air samples from various BEs, including a large urban building, a medical center, a house, and a pier (Yooseph et al., 2013). This study highlights that indoor air is mainly rich in human-associated bacterial DNA, principally bacteria of the *Pseudomonas* genus, commonly found on human skin, whereas outdoor air exhibited a more diverse mix of DNA fragments from the environment, plants, and animals. Notably, outdoor air in New York City showed an abundance of ARGs, like β-lactamases and tetracycline (Gilbert and Stephens, 2018).

The BE microbiome was investigated also at a larger spatial level. For instance, the microbiome and resistome of public transit air of multiple cities across three continents (Asia, Europe, and United States) were evaluated by shotgun metagenomics combined with standardized air sampling and bioinformatics methodologies (Leung et al., 2021). Furthermore, a global study spanning 60 cities over 3 years provided insights into the ecology, virulence, and ARGs of city-specific microbial communities (Danko et al., 2021). Led by the Metagenomics and Metadesign of Subways and Urban Biomes International Consortium, the research involved a comprehensive analysis of surface microbiomes and resistomes across various public transit systems worldwide, examining microbial strain profiles, ARG markers, functional, and genetic characteristics (MetaSUB International Consortium, 2016).

Merino et al. (2019) conducted a comprehensive genomic analysis exploiting full genome sequencing to understand the bacterial lifestyles within BEs. They distinguish BE bacteria from “others” for their genomic features such as larger genomes, and higher GC content. This suggests a potential advantage in gene expression levels, deriving from the long-term association with humans.

Even the meta-analysis studies of BE microbiota have started to propagate. An example is the pangenomes meta-analysis of two frequent members of the indoor microbiome, *B. cereus*, and *S. aureus*, deriving from whole-genome sequencing (WGS) data of strains isolated from the International Space Station (ISS; i.e., a model BE), BEs on Earth, soil, and humans (Blaustein et al., 2019). The study showed a significant correlation between the genomic features of the two taxa and their origin (either Earth or ISS), suggesting complex biological processes for potential niche adaptations that do not impact human health.

However, the attractive approach of combining data from different data sets and meta-analysis studies for better comprehension of BE microbial communities has often had multiple limitations. First, the varied nature of the BE microbiome complicates efforts to standardize methods and the sampling procedure from different matrices, especially indoors. Second, this approach lacks appropriate reference controls (Adams et al., 2015). Moreover, these surveys are often limited in sample number, geographically, culture/WGS data for single strains, and potential sources and manipulation of BE (Blaustein et al., 2019). Therefore, standardizing data collection and description methods would streamline comparisons among studies and foster collaborations (Adams et al., 2015). In this scenario, Gomez-Silvan et al. (2018) evaluated methodological variations using a mock BE microbial community. They found significant technique-and biological-based differences in both nucleic acid processing and analysis methods. The study suggested performing nucleic acid extraction within a week of sampling and storing them appropriately to minimize technical disparities. They also noted variations in DNA/RNA co-extraction efficiency for different microbes. Biological variation showed large discrepancies between DNA and RNA analyses in taxonomy, and microbial associations. The authors recommended relying on rRNA from a residential BE microbiome to identify the potentially viable portion of the microbial community, even including dead and inactive cells, as this approach offers ecologically relevant insights into indoor microbial dynamics.

Despite the advancements in metagenomic approaches, limitations persist due to technological sequencing issues, impacting the accuracy and comprehensiveness of BE bacterial community analysis. While whole genomes offer richer information compared to marker genes, challenges remain in accurately assembling genomes, especially for low-abundance organisms (Hester et al., 2019; Ayling et al., 2020). This hampers the interpretation of population-level genetic variation. However, the availability of long-read sequencing holds promises in addressing these technological limitations. Another key point is the accuracy of automated annotation of genetic elements in genomes. Indeed, database quality influences the results of survey studies and meta-analyses (Hester et al., 2019). For instance, the study of metabolic overlap (MO) among microbial communities on a genome-scale across various ecosystems involved nearly 1,000 studies to develop a new metric for microbial functional redundancy, potential metabolic competition, and cooperation. MO varied across environments, with extreme and aquatic environments exhibiting the highest MO, while communities associated with animal hosts, the built environment, and soil had the lowest MO. Moreover, MO between species can be influenced by both genome size and phylogenesis, indicating potential metabolic interactions between species in an ecosystem (Hester et al., 2019).

Regardless these meta-analysis studies and mock community assessments are valuable in defining the accuracy, sensitivity, and specificity of microbial classifiers, a complex BE microbial community could provide a more realistic understanding of biological classifiers and mechanistic relationships (Lee et al., 2023). However, surveys regarding a genomic-functional reconstruction often lack information about gene transcription, translation, and protein affinity and activity could be limiting (Hester et al., 2019). Therefore, complementary techniques, such as transcriptomics, proteomics, and metabolomics could fulfill the current knowledge.

An innovative approach to comprehensively studying environmental exposures and their effects on health is the exposome study. For example, a multi-center group of 1,301 mother–child pairs associated with more than 100 chemicals, outdoor, social, and lifestyle exposures were evaluated during pregnancy and childhood with multi-omics profiles (Maitre et al., 2022). It shows potential epigenetic biomarkers and biological responses correlated to the diverse origin of exposure, enhancing understanding of disease mechanisms. The transcriptomic approach evidenced the complexity of transcriptional regulation insinuating that different molecular levels can picture only a portion of the exposome effects, and the metabolomics approach accurately portrayed dietary sources and the potential gut microbial effect of exposures (Maitre et al., 2022).

Probably expanding such investigations to diverse BEs, targeting a wider range of sources, locations, populations, and biological markers could provide deeper insights into BE microbiomes and their impacts on health. Nevertheless, recently the concept of “urbanome” (i.e., the genome of the city) was proposed to address the complexity of BE and design effective interventions for urban sustainability (Morawska et al., 2022). By quantifying behavioral and health outcomes in urban areas, the urbanome assessment could inform policies and interventions to maximize benefits and minimize problems in future cities.

## 4 Native niches of BE most represented bacteria

**Figure 4:**
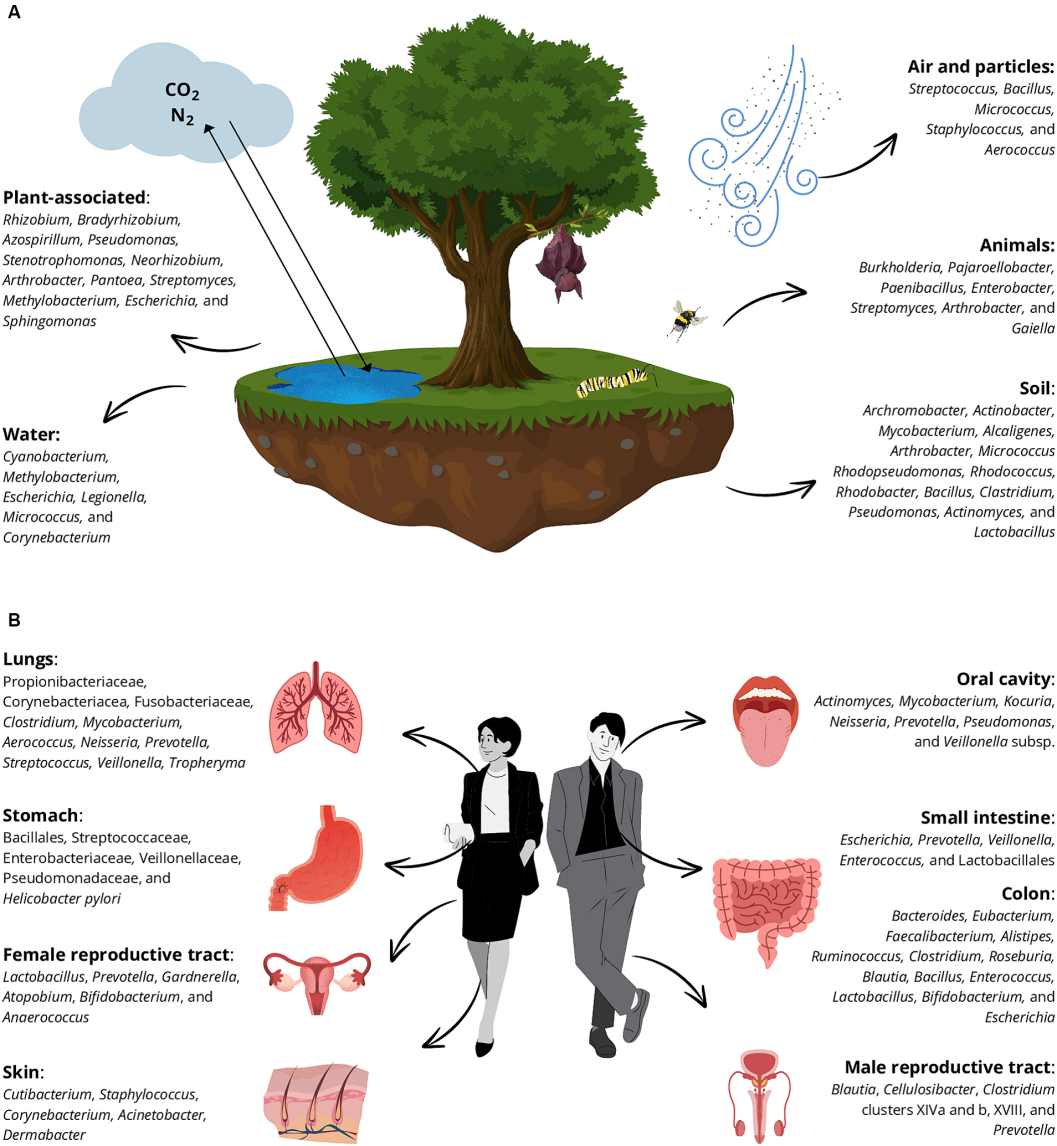
Principal bacterial genera that belong to the natural environments **(A)** and human habitats **(B)** are distributed in diverse native niches. The main niches are plant-associated environments, water systems, soil, animals, and air and particles in **(A)** and different human compartments **(B)**: lungs, stomach, male and female reproductive tracts, skin, oral cavity, small intestine, and colon.

Bacteria are not mere inhabitants of places and bodies but mediate numerous processes that affect mass and energy flows within each system (King, 2014). To have a comprehensive vision of bacterial roles, it is fundamental to have a broad knowledge of features of the bacterial natural niches, the inhabitants of diverse environments and consequently understanding which bacteria manage to survive in the BE and to interact with both humans and the environment. Recent research has shown that microbes exhibit biogeographic distribution and are also dispersal-limited leading to the bacterial denomination of “invaders” when they colonize a new environment, challenging the notion that “everything is everywhere but the environment selects” (Mallon et al., 2015). This is true even if we talk about bacteria associated with humans, as different species tend to colonize different body districts (McCallum and Tropini, 2024). Among the bacteria considered in this review, around one-third are associated only with the environment (in the strict sense), one-third with humans, and the others cannot be solely associated with a specific compartment (Supplementary Table S1; Figure 4).

### 4.1 Natural environments

Among natural environments, the soil is the major source of microorganisms in terrestrial ecosystems (Banerjee and van der Heijden, 2023). Soil bacteria form complex communities together with archaea, fungi, protozoa, and viruses, collectively known as soil microbiota. They principally belong to Pseudomonadota, Actinomycetota, Cyanobacteriota, and Acidobateriota (formerly Acidobacteria) phyla (Figure 4A) (Banerjee and van der Heijden, 2023). Importantly, this plethora of microorganisms plays key roles in the environment, such as nutrient (carbon, nitrogen, and phosphorus) cycling, organic matter decomposition, soil structure definition, plant disease suppression, and plant productivity support (Gougoulias et al., 2014; Coban et al., 2022). Various bacterial groups, including plant growth-promoting rhizobacteria, nitrogen-fixing bacteria, and *Cyanobacteria* contribute to soil fertility and plant growth. Among others, an example is the *Rhizobium* genus commonly constituted by nitrogen-fixing bacteria whose presence in the BE indoors can be exploited as a “sensor-bacterium” to figure out an exposure to the outdoor environment, to reconstruct the “age” of a certain building, or to evaluate the ventilation of the construction itself, ideally abolishing the internal-external difference (Cao et al., 2021).

Regarding the organic matter degraders, bacteria belonging to the *Archromobacter*, *Alcaligenes*, *Arthrobacter*, *Rhodopseudomonas*, *Rhodobacter*, *Bacillus*, and *Lactobacillus* genera are the most well-known xenobiotic degraders from soil, thus also used for microbial-based cleaning products for indoor surfaces (Arvanitakis et al., 2018; D’Accolti et al., 2019).

Despite abiotic restraints such as low availability of water, organic carbon, and nitrogen substrates, pH, temperature, redox state, and biotic limitations such as competition, predation, and negative interactions with soil flora and fauna make difficult for bacteria to colonize the soil, they are necessary for the formation and shaping of soil microbiota composition (Tkacz et al., 2020). Additionally, the microbiota has usually strong intercommunications with the soil biotic fraction including plants (Rossi et al., 2021; Banerjee and van der Heijden, 2023), and animals (Kikuchi et al., 2007; Hannula et al., 2019). For instance, they contribute to soil microbiota diversity through activities like geophagy or increasing in potentially harmful bacteria due to animal excretion (Li A. et al., 2021; Zhang et al., 2022; Banerjee and van der Heijden, 2023). Concerning harmful bacteria, they evolve some strategies to survive in the soil, such as sporulation or the viable but not culturable state making them resilient in soil and potentially hazardous to human health (Browne et al., 2017). For example, urban and non-urban soil can also harbor harmful pathogens like *Clostridium difficile* (Khun et al., 2023), *S. aureus* (Li et al., 2020), and *Burkholderia cepacea* (Miller et al., 2002) which can cause infections in humans.

Overall, understanding soil microbiota and its interactions with the environment is crucial for maintaining soil quality and preventing the spread of pathogens.

Air, seemingly inhospitable, is actually teeming with life both indoors and outdoors. Though it might not seem conducive to microbial life due to harsh conditions such as drying, UV radiation low nutrients, and temperatures, air harbors diverse microbial communities (Naumova and Kabilov, 2022). Among the diverse bacterial sources, the surrounding environments, and human activities, the soil is a major source of airborne bacteria, especially when rainfall aerosolizes bacterial-rich droplets (Joung et al., 2017). Wind and plants also contribute to airborne bacteria, along with animals through respiration and their microbiota (Xie et al., 2021). In the atmosphere, bacteria are widespread and can influence cloud formation and chemistry. Some airborne pathogens pose risks to human health, causing diseases and allergies. However, it is not certain if these bacteria are an ecological community or simply a pool of microbes passively accumulated (Gandolfi et al., 2013; Gong et al., 2020). The possible pathogens present in the near-surface atmosphere can have effects on human health, such as infectious diseases, toxicity, allergies, and even cancer (Gong et al., 2020).

The composition of airborne microbes varies by season and region, affected by factors like temperature, humidity, and particulate matter concentrations (Moelling and Broeker, 2020). Generally, 70–90% of cultured airborne bacteria are Gram-positive (mainly *Bacillus*, *Micrococcus*, and *Staphylococcus*). A notable exception reported in the literature is the urban area of Marseilles (France) because 60% of analyzed bacteria were Gram-negative. The situation reverses if sequencing techniques are used, highlighting the presence of Proteobacteria (mainly *Burkholderiales* and *Moraxellaceae*) (Gandolfi et al., 2013).

Indoor air has a lower diversity of microbes compared to outdoors, with a higher proportion originating from human respiratory systems and skin shedding, including Pseudomonadota, Bacillota, Actinomycetota, and Bacteroidota (Hayleeyesus and Manaye, 2014; Miletto and Lindow, 2015; Moldoveanu, 2015; Prussin and Marr, 2015; Leung and Lee, 2016; Dai et al., 2017). Building design, ventilation, airflow direction, temperature, and relative humidity play a relevant role in indoor air quality (Dai et al., 2017). Indeed, proper ventilation is essential for mitigating health risks associated with indoor pollutants and pathogens like *Legionella pneumophila* (Li S. et al., 2021; Gea-Izquierdo et al., 2023).

### 4.2 Human beings

The human microbiota is composed of bacteria, archaea, viruses, and eukaryotes, both inside and outside the body (Figure 4B) (Ogunrinola et al., 2020). In the last decade, there was a paradigm shift leading to the consideration of eukaryotes as meta-organisms inseparable from their microbiota, impacting human physiology profoundly (Berg et al., 2020). Functionally, the microbiota acts as a “hidden organ,” influencing metabolic and immune functions (Hou et al., 2022). It evolves continually in response to various host factors such as age, nutrition, and lifestyle. The immune system interacts with the microbiota, responding to pathogens and diversity loss (dysbiosis) (Berg et al., 2020). The microbiota is not uniformly distributed but varies across different body sites, each with specific environmental conditions creating distinct niches. For instance, the gastrointestinal (GI) tract harbors mainly facultative and strict anaerobes, while aerobic bacteria dominate exposed compartments like the respiratory tract and skin (Berg et al., 2020; Ogunrinola et al., 2020).

The gut microbiota, the largest bacterial community in the body, plays a crucial role in maintaining human health by aiding in digestion and protecting against pathogen invasion. It is interconnected with other microbial communities like the vaginal and oral microbiota, influencing each other’s composition and function. The oral cavity, the entry point for food digestion, hosts diverse microbial environments rich in different microbial taxa (Ruan et al., 2020; Hou et al., 2022). Moving through the GI tract, distinct microbiota populations thrive in various segments due to differences in pH, bile content, and nutrient availability, including bacteria belonging to the Bacillales, Steptococcaceae, Enterobacteriaceae, Veillonellaceae, and Pseudomonadaceae, as well as the pathogen *Helicobacter pylori* (Ruan et al., 2020). The colon, particularly, houses a complex microbiota responsible for fermenting complex carbohydrates and absorbing water and minerals including microbes shared by all the adults, belonging to the genera *Bacteroides*, *Eubacterium*, *Faecalibacterium*, *Alistipes*, *Ruminococcus*, *Clostridium*, *Roseburia*, and *Blautia*. Certain bacterial genera, such as *Bacillus*, utilize spore-forming capabilities for survival and transmission of pathogenesis. Some *Bacillus* species are pathogens, while others, like *B. clausii* and *B. coagulans* as well as *Enterococcus*, *Lactobacillus*, and *Bifidobacterium* are utilized for their beneficial roles as probiotics (Presti et al., 2015; Elshaghabee et al., 2017; Sorbara and Pamer, 2022).

The skin, acting as a physical and immunological barrier, has a dry surface, with round 5.6 pH, and a temperature lower than the inside of the body (Skowron et al., 2021). It is sprinkled with glands and hair follicles that create different bacterial niches characterized by different conditions of temperature, exposure to external agents, and molecules composing skin cells and secretions (Hou et al., 2022). Dominant resident bacteria like *Cutibacterium*, *Staphylococcus*, and *Corynebacterium* exert protective functions through various mechanisms, preventing pathogen colonization and they are influenced by hygiene habits (Hospodsky et al., 2012; Kelley and Gilbert, 2013; Adams et al., 2015; Prussin and Marr, 2015; Wilkins et al., 2016; Gilbert and Stephens, 2018; Merino et al., 2019; Rai et al., 2021; Skowron et al., 2021).

In the respiratory tract, the upper and lower regions create ecological niches for microorganisms, although until not long ago, the lungs were thought to be sterile. The oropharynx serves as the main source of adult lung microbiota, dominated by Bacillota and Bacteroidota (Man et al., 2017). The lung microbiota is composed of a transient community rather than a resident community. Indeed, the balance of microbial immigration and elimination mirrors an ecological equilibrium. Besides, the microbiota has a protective action for the host, using active mechanisms of pathogen exclusion (Man et al., 2017).

The reproductive tract microbiota differs between sexes and can serve as a fingerprint in BEs (Prussin and Marr, 2015). Female reproductive tract microbiota, rich in *Lactobacillus* species, maintains vaginal homeostasis, while male genital tract microbiota shows fewer studies since sampling is highly invasive and they include *Blautia*, *Cellulosibacter*, and *Clostridium*, and *Prevotella* (Moreno and Simon, 2018; Zuber et al., 2023).

## 5 Built environment microbiota remodulation as a new paradigm for human health and prevention methods against diseases

Understanding the microbiology of the BE could be a useful tool for the remodulation of the BE microbiota in the vision of preventing emerging diseases and preserving human health for building materials, indoor plants, and service quality. Bacteria reveal dual impacts, both positive and negative, thus distinguishing between beneficial and harmful bacteria (Supplementary Table S2).

The beneficial bacteria can exert their positive functions toward human well-being like probiotics, the environmental quality, like decomposer and biodegrading bacteria, and in favor of plant growth.

Probiotics including *Enterococcus*, *Lactobacillus*, and *Bifidobacterium* are known to enhance human health by maintaining a microbial balance, strengthening the mucosal intestinal barrier, detoxifying toxic compounds, and the release of helpful molecules (Hoisington et al., 2015; Presti et al., 2015; Feng et al., 2018; De Giani et al., 2022). Within the BE in places like hospitals and homes, a “probiotic approach” can promote and facilitate the growth of beneficial microorganisms. For example, mycobacteria found indoors in water sources can confer protection from asthma, have antidepressant-like behavioral effects, reduce anxiety, and improve cognitive function (Stamper et al., 2016). Recently, *Lactobacillus* bacteria have even been employed in microbial-based cleaning products for their xenobiotic degrading capacities (Adams et al., 2015; Rai et al., 2021). Besides Lactobacilli, other organic matter degraders including bacteria belonging to the *Alcaligenes*, *Arthrobacter*, *Rhodopseudomonas*, *Rhodobacter*, and *Bacillus* genera are found in BE, are used for cleaning products to break down waste materials and maintain healthy indoor environments (Arvanitakis et al., 2018; D’Accolti et al., 2019). Related to this, bacteria able to degrade or detoxify pollutants are notable for bioremediation processes, including *Actinomyces,* known for the degradation of organic plant material, lignin, and chitin, forming compost (Hewitt et al., 2012; Wilkins et al., 2016), *Streptomyces*, *Delftia*, *Ralstonia,* and *Rhodococcus*, well-recognized contaminant-degraders, metabolizing several hydrocarbons, pesticides, and polymers (Prussin and Marr, 2015; Merino et al., 2019; Zampolli et al., 2019; Brevik et al., 2020; Cao et al., 2021; Peng et al., 2022; Zampolli et al., 2022). Indirectly beneficial for human and environmental prosperity are mutualistically plant growth-promoting (Rossi et al., 2021; Coban et al., 2022), including *Azospirillum brasilense*, *Pseudomonas putida*, and *Arthrobacter globiformis*, nitrogen-fixing bacteria like *Bradyrhizobium*, *Neorhizobium*, and *Rhizobium* species (Hewitt et al., 2012; Kelley and Gilbert, 2013; Adams et al., 2017; Merino et al., 2019; Cao et al., 2021; Rai et al., 2021; Rossi et al., 2021; Coban et al., 2022) or antagonizing soil pathogens for plant survival, such as such as *Methylobacterium* spp., and *Sphingomonas* spp. (Xiong and Lu, 2022) which are also found indoors.

Unfortunately, harmful microorganisms like pathogens or biofilm-forming bacteria can thrive in BEs, especially with poor hygiene practices. For instance, the presence of *Bordetella*, an opportunistic pathogen often associated with respiratory illnesses, raises concerns about the potential transmission of infectious diseases in public spaces, like shopping centers (Gilbert and Stephens, 2018). Likewise, *Acinetobacter* spp., primarily found in hospitals, but was also detected in indoor air (shopping centers), is notorious for causing infections to diverse human compartments (Gauzere et al., 2014). Additionally, the presence of commensal bacteria like *Bacillus* spp. (*B. anthracis*, and *B. cereus*), *Staphylococcus* spp. and *Streptococcus* spp. on human skin and mucous membranes is generally harmless in healthy individuals. However, they can cause respiratory infections under certain circumstances, especially in immunocompromised individuals (Hospodsky et al., 2012; Kelley and Gilbert, 2013; Adams et al., 2015; Prussin and Marr, 2015; Leung and Lee, 2016; Wilkins et al., 2016; Adams et al., 2017; Gilbert and Stephens, 2018; Merino et al., 2019; Cao et al., 2021; Rai et al., 2021; Panthee et al., 2022), as well as *B. cepacea* (Miller et al., 2002). Another genus exhibiting members with a dual nature, opportunistic pathogens, and commensal bacteria is *Mycobacterium* spp. (Gauzere et al., 2014; Adams et al., 2017; Browne et al., 2017; Gilbert and Stephens, 2018; Merino et al., 2019; Brevik et al., 2020; Horve et al., 2020). They adopt the strategy of being in a dormancy state to be viable but not culturable (Browne et al., 2017; Li et al., 2020). Other human pathogens belong to *Clostridium perfrigens*, *C. botulinum*, and *C. tetani* which can be transmitted via skin trauma (Brevik et al., 2020). The emergence of *Stenotrophomonas* spp. as a potential opportunistic pathogen indoors suggests a need for further investigation into its transmission routes and associated health risks (Cao et al., 2021).

Certain species of *Legionella* (*L. pneumophila*), and *Pseudomonas aeruginosa* present a significant public health concern since the bacteria of this species can form biofilms in water or ventilation systems, and with inadequate sanitation practices can thrive and spread infectious diseases (Gilbert and Stephens, 2018; Brevik et al., 2020; Horve et al., 2020). Similarly, airborne associated bacteria are a great issue, such as *Vibrio harveyi* a human pathogen detected in hospitals (Rai et al., 2021).

The contamination due to excretion potentially increases harmful bacteria and indicators of fecal contamination like *C. difficile, Escherichia coli*, and *Enterococcus* are widespread (Browne et al., 2017; Gilbert and Stephens, 2018; Merino et al., 2019; Horve et al., 2020; Rai et al., 2021; Khun et al., 2023; Leri and Khan, 2023). Pathogenic strains such as *Salmonella* and *Shigella* spp. pose risks through the fecal-oral route, often transmitted via contaminated food or water sources (Prussin and Marr, 2015; Browne et al., 2017; Brevik et al., 2020).

Some microorganisms produce allergens that can trigger allergic reactions in sensitive individuals. For example, dust mites and their waste products can exacerbate allergies and asthma symptoms in indoor environments. In addition, Horve et al. (2020) reported the presence of *Cyanobacterium* in office buildings, where they are responsible for the prevalence of allergic reactions. Other candidates associated with asthma and allergies are *Listeria monocytogenes* and *Bacillus* spp. (Dai et al., 2017).

In order to prevent harmful bacteria colonization, a general approach is to make a specific environment as hostile to microbial life as possible (Gilbert and Stephens, 2018), thus, regular cleaning, proper ventilation, and temperature and moisture control are essential strategies for managing microbial populations in indoor spaces. Specifically, building design is relevant, considering physical barriers, the type of ventilation (mechanical or natural), the direction of airflow, the presence of mechanical filters, electrostatic precipitators, non-thermal plasma air purifiers, photocatalytic oxidation system, or UV disinfection (Dai et al., 2017). For surfaces, biocides (permanently bound or released), anti-adhesive, antimicrobial light, and touch-free solutions can be considered. For controlling plumbing pathogens, water pipeline design and configuration, choice of water outlets, the materials used in contact with water, filtration, disinfection by chlorine-based chemicals, UV light, ozone, and copper-silver ionization, temperature, and flow regulation should be taken into consideration (Salonen et al., 2023) (Supplementary Table S2).

## 6 Conclusion

The microbiology of the BE is a research area in the early days due to the complexity of this environment and the interactions within it. The comprehension of which BE microbiomes, the microbial combinations, their main roles, and characteristics may affect human health is extremely challenging. Nevertheless, evidence that microbial exposure can have beneficial health impacts has increased the interest in managing and manipulating BEs to revise such impacts.

The development of molecular technologies for the analysis of BE microbiome samples has significantly favored our interpretation and perception of BE microbes. Indeed, the sequence-based approaches principally served for taxa reconstruction. However, knowledge about the functions, expression levels, and viability of the vast numbers of microorganisms present in the BE, are still guessed. Furthermore, multidisciplinary surveys are very much needed to address the maintenance of the equilibrium of environmental and human microbiome shifting considering the natural functions of microorganisms in their native niches. A substantial effort could be focused on the preservation of the beneficial microorganisms, their functions, and interactions (with each other and with the surroundings) as a reward for the relationship between the environment and humans and their microbiota, and ultimately human health. In addition, proper environmental management and the improvement of lifestyle habits are urgent needs in the current global scenario to restore an urbanization level more affordable and sustainable.

## Author contributions

JZ: Conceptualization, Validation, Writing – original draft, Writing – review & editing. AG: Conceptualization, Visualization, Writing – original draft. MR: Writing – original draft. MF: Validation, Visualization, Writing – review & editing. PG: Funding acquisition, Project administration, Resources, Supervision, Writing – review & editing.
