# Supplementary material for: Who inhabits the built environment? A microbiological point of view on the principal bacteria colonizing our urban areas
Source: Front Microbiol. 2024 May 28;15:1380953. doi: 10.3389/fmicb.2024.1380953 (PMC11165352; doi:10.3389/fmicb.2024.1380953)
Supplement: Supplementary file 2 [file Table_2.DOCX]

**Table S2.** Beneficial and harmful bacteria of BE distinguishing their positive role, the adverse effects, and the eventual preventive methods.

| **Genus/Species** | **Location in the BE** | **Human or environment association** | **Human tract infection and associated diseases** | **Infection-prevention or antimicrobial methods** | **References** |
| --- | --- | --- | --- | --- | --- |
| **Beneficial bacteria** | | | | | |
| Probiotics | | | | | |
| *Lactobacillus* spp. | Homes, office, indoor air, surface dust, subway, classroom (also university), wastewater, sewage | Human | - | - | Hoisington et al., 2015, Presti et al. 2015, Feng et al. 2018, De Giani et al. 2022 |
| *Bifidobacterium* spp. | Wastewater, wastewater treatment  plants | Human | - | - | Hoisington et al., 2015, Presti et al. 2015, De Giani et al. 2022 |
| *Enterococcus* spp. | Indoor, hospital, subway, park | Human, Airborne | - | - | Hoisington et al., 2015, Presti et al. 2015, De Giani et al. 2022 |
| *Mycobacterium* spp. | Indoor (air and surfaces), gym shopping center, water heaters and kitchen, hospital, subway | Human, Soil | - | - | Gauzere et al. 2014, Stamper et al. 2016, Browne et al. 2017, Adams et al. 2017, Gilbert et al. 2018, Merino et al. 2019, Brevik et al. 2020, Horve et al. 2020 |
| Decomposer bacteria | | | | | |
| *Alcaligenes* | Hospital, indoor | Soil, Water | - | - | Arvanitakis et al. 2018 |
| *Arthrobacter* | Indoor, dust, subway | Soil | - | - | Merino et al. 2019, Peng et al. 2022 |
| *Bacillus* spp. | Indoor, office, museum, hospital, bathroom, subway, gym | Human, Droplet-associated, Soil | - | - | Hewitt et al. 2012, Kelley et al. 2013, Gauzere et al. 2014, Prussin et al. 2015, Leung et al. 2016, Gilbert et al. 2018, Merino et al. 2019, Brevik et al. 2020, Horve et al. 2020, Egan et al. 2021 |
| *Clostridium* spp. | Indoor, kitchen, subway | Human, Soil | - | - | Gauzere et al. 2014, Gilbert et al. 2018, Merino et al. 2019, Brevik et al. 2020, Egan et al. 2021 |
| *Pseudomonas* spp. | Outdoor, indoor air, modern building, hospital, family home, pier, office, museum, kitchen | Environment, Human, Soil, Plant | - | - | Hewitt et al. 2012, Gauzere et al. 2014, Adams et al. 2015, Prussin et al. 2015, Gilbert et al. 2018, Rai et al. 2020, Brevik et al. 2020, Rai et al. 2020 |
| *Rhodopseudomonas* | Indoor | Soil, Sediments, Water | - | - | Horve et al. 2020 |
| *Rhodobacter* | Household's air | Environment, Freshwater | - | - | Wilkins et al. 2016 |
| Bioremediation bacteria | | | | | |
| *Actinomyces* spp. | Office, house surface | Soil, Human | - | - | Hewitt et al. 2012, Wilkins et al. 2016 |
| *Delftia* spp. | Hospital | Soil | - | - | Merino et al. 2019 |
| *Ralstonia* spp. | Plastic fiber surface in indoor, hospital, indoor air | Animals | - | - | Merino et al. 2019, Peng et al. 2022 |
| *Rhodococcus* spp. | Indoor old building, university | Environment, Soil, Water | - | - | Cao et al. 2021, |
| *Stenotrophomonas* spp. | Indoor new building, university, hospital, subway | Environment, plants, soils | - | - | Merino et al. 2019, Cao et al. 2021 |
| *Streptomyces* | Indoor | Human, Soil, Plant (decaying vegetation) | - | - | Prussin et al. 2015, Brevik et al. 2020 |
| Plant growth-promoting and nitrogen-fixing bacteria | | | | | |
| *Bradyrhizobium* spp. | Office, indoor wall surfaces, bathroom | Human, Rhizosphere, Soil | - | - | Hewitt et al. 2012, Kelley and Gilbert 2013, Merino et al. 2019 |
| *Neorhizobium* spp. | Kitchen | Plant | - | - | Rai et al. 2020, Adams et al. 2017 |
| *Rhizobium* spp. | Indoor building, university | Rhizosphere, Soil | - | - | Cao et al. 2021, Rossi et al. 2021, Coban et al. 2022 |
| *Azospirillum brasilense* | Indoor | Rhizosphere, Soil | - | - | Xiong and Lu 2022 |
| *Pseudomonas putida* | Outdoor, indoor air, modern building, hospital, family home, pier, office, museum, kitchen | Environment, Human, Soil, Plant | - | - | Rossi et al. 2021 |
| *Arthrobacter globiformis* | Indoor, subway | Soil | - | - | Merino et al. 2019, Rossi et al. 2021, Peng et al. 2022 |
| *Methylobacterium* spp. | Household's air, kitchen water, bathroom, hospital, subway, office, dust, university | Environment, Soil, Water | - | - | Xiong and Lu 2022 |
| *Sphingomonas* spp. | Outdoor, office, museum, hospital, bathroom, subway, university classroom, hotel, gym, park, dust | Environment, Human | - | - | Kelley et al. 2013, Gauzere et al. 2014, Adams et al. 2015, Rai et al. 2020, Merino et al. 2019, Xiong and Lu 2022 |
| **Harmful bacteria** | | | | | |
| Human pathogenic bacteria | | | | | |
| *Acinetobacter* spp. | Kitchens, outdoor, indoor, hospital, office, museum, surfaces, university classroom, bathroom, subway, gym | Soil, Human | Nosocomial infections (respiratory tract, skin, and gastrointestinal tract) | Ventilation, the direction of airflow, mechanical filters, electrostatic precipitators, non-thermal plasma air purifiers, photocatalytic oxidation system, UV disinfection, and surface biocides, anti-adhesive, antimicrobial light, touch-free solutions | Gauzere et al. 2014, Adams et al. 2015, Prussin et al. 2015, Leung et al. 2016, Wilkins et al. 2016, Gilbert et al. 2018, Merino et al. 2019, Rai et al. 2020, Cao et al. 2021 |
| *Bacillus cereus* and *anthracis* | Indoor, office, museum, hospital, bathroom, subway, gym | Human, Droplet-associated, Soil | Respiratory and gastrointestinal infections | Ventilation, the direction of airflow, mechanical filters, electrostatic precipitators, non-thermal plasma air purifiers, photocatalytic oxidation system, UV disinfection, and surface biocides, anti-adhesive, antimicrobial light, touch-free solutions | Hewitt et al. 2012, Kelley et al. 2013, Gauzere et al. 2014, Prussin et al. 2015, Leung et al. 2016, Gilbert et al. 2018, Merino et al. 2019, Brevik et al. 2020, Horve et al. 2020, Egan et al. 2021 |
| *Bordetella* spp. | Shopping center | Human | Respiratory tract, whooping cough | Ventilation, the direction of airflow, mechanical filters, electrostatic precipitators, non-thermal plasma air purifiers, photocatalytic oxidation system, UV disinfection | Gilbert et al. 2018 |
| *Burkholderia* spp. | Office, indoor, bathroom, hospital, park, gym | Human, Airborne, soil | Respiratory infections and pneumonia | Ventilation, the direction of airflow, mechanical filters, electrostatic precipitators, non-thermal plasma air purifiers, photocatalytic oxidation system, UV disinfection | Miller et al. 2002, Kelley et al. 2013, Prussin et al. 2015, Merino et al. 2019, Rai et al. 2020, Brevik et al. 2020 |
| *Mycobacterium* spp. | Indoor (air and surfaces), gym shopping center, water heaters and kitchen, hospital, subway | Human, Soil | Respiratory infections and pulmonary diseases | Ventilation, the direction of airflow, mechanical filters, electrostatic precipitators, non-thermal plasma air purifiers, photocatalytic oxidation system, UV disinfection, surface biocides, anti-adhesive, antimicrobial light, touch-free solutions | Gauzere et al. 2014, Stamper et al. 2016; Browne et al. 2017, Adams et al. 2017, Gilbert et al. 2018, Merino et al. 2019, Brevik et al. 2020, Horve et al. 2020 |
| *Staphylococcus* spp. | Indoor, office, air of healthy office building, hospital, railway, subway, gym, university classroom, shower stall, ventilation duct supply, dust, outdoor, hotel | Human | Respiratory and gastrointestinal infections | Ventilation, the direction of airflow, mechanical filters, electrostatic precipitators, non-thermal plasma air purifiers, photocatalytic oxidation system, UV disinfection, and surface biocides, anti-adhesive, antimicrobial light, touch-free solutions | Hospodsky et al. 2012, Kelley et al. 2013, Adams et al. 2015, Prussin et al. 2015, Wilkins et al. 2016, Leung et al. 2016, Adams et al. 2017, Gilbert et al. 2018, Merino et al. 2019, Rai et al. 2020, Cao et al. 2021, Panthee et al. 2022 |
| *Stenotrophomonas* spp. | Indoor new building, university, hospital, subway | Environment, plants, soils | Respiratory tract infections | Building design, physical barriers, regular cleaning, proper ventilation, and temperature and moisture control | Merino et al. 2019, Cao et al. 2021 |
| *Streptococcus* spp. | Bathroom, hospital, subway, office, indoor air, ventilation duct supply, dust, university classroom, household air, kitchen, indoor wall surface, hotel, gym, | Human, Airborne | Respiratory infections and pneumonia | Ventilation, the direction of airflow, mechanical filters, electrostatic precipitators, non-thermal plasma air purifiers, photocatalytic oxidation system, UV disinfection, and surface biocides, anti-adhesive, antimicrobial light, touch-free solutions | Hospodsky et al. 2012, Kelley et al. 2013, Adams et al. 2015, Prussin et al. 2015, Wilkins et al. 2016, Adams et al. 2017, Gilbert et al. 2018, Merino et al. 2019, Rai et al. 2020 |
| *Vibrio harveyi* | Indoor | Airborne, Marine environment | Gastrointestinal tract infections | Ventilation, the direction of airflow, mechanical filters, electrostatic precipitators, non-thermal plasma air purifiers, photocatalytic oxidation system, UV disinfection | Rai et al. 2020 |
| Biofilm-forming bacteria | | | | | |
| *Legionella* spp. and *L. pneumophila* | Indoor, water and ventilation system | Droplet-associated, Soil, Water | Respiratory tract infections and pneumonia | Water pipeline configuration, choice of water outlets, the materials used in contact, filtration, disinfection by chlorine-based chemicals, UV light, ozone, and copper-silver ionization, temperature, flow regulation | Gilbert et al. 2018, Brevik et al. 2020, Horve et al. 2020 |
| *Pseudomonas aeruginosa* | Outdoor, indoor air, modern building, hospital, home, pier, office, museum, kitchen | Environment, Human | Respiratory tract infections and pulmonary diseases | Ventilation, the direction of airflow, mechanical filters, electrostatic precipitators, non-thermal plasma air purifiers, photocatalytic oxidation system, UV disinfection | Gilbert et al. 2018, Brevik et al. 2020, Horve et al. 2020 |
| Fecal contamination indicators | | | | | |
| *Escherichia* spp. | Indoor, kitchen, bathroom, hospital, subway, gym | Human, Water | Gastrointestinal and urinary tract infections, bowel diseases, enterocolitis | Water pipeline configuration, choice of water outlets, the materials used in contact, filtration, disinfection by chlorine-based chemicals, UV light, ozone, and copper-silver ionization, temperature, flow regulation, and surface biocides, anti-adhesive, antimicrobial light, touch-free solutions | Horve et al. 2020, Merino et al. 2019, Leri et al. 2023 |
| *Enterococcus spp.* | Indoor, hospital, subway, park | Human, Airborne | Gastrointestinal tract infections, bowel diseases, enterocolitis | Water pipeline configuration, choice of water outlets, the materials used in contact, filtration, disinfection by chlorine-based chemicals, UV light, ozone, and copper-silver ionization, temperature, flow regulation, and surface biocides, anti-adhesive, antimicrobial light, touch-free solutions | Gilbert et al. 2018, Browne et al. 2017, Merino et al. 2019, Rai et al. 2020, Leri et al. 2023 |
| *Clostridium difficile, C. perfringens, C. tetani, C. botulinum* | Indoor, kitchen, subway | Human, Soil | Inflammatory bowel disease, tetanus, and botulism | Surface biocides, anti-adhesive, antimicrobial light, touch-free solutions | Gauzere et al. 2014, Gilbert et al. 2018, Merino et al. 2019, Brevik et al. 2020, Egan et al. 2021 |
| *Salmonella* spp. | Plumbing system | Human, Stool, Soil | Gastrointestinal tract infections, bowel diseases, enterocolitis | Water pipeline configuration, choice of water outlets, the materials used in contact, filtration, disinfection by chlorine-based chemicals, UV light, ozone, and copper-silver ionization, temperature, flow regulation | Prussin et al. 2015, Browne et al. 2017, Brevik et al. 2020 |
| Allergens | | | | | |
| *Cyanobacterium* | Office, indoor | Water | Respiratory tracts | Ventilation, the direction of airflow, mechanical filters, electrostatic precipitators, non-thermal plasma air purifiers, photocatalytic oxidation system, UV disinfection | Horve et al. 2020 |
| *Listeria monocytogenes* | Indoor, dust | Soil, Water | Respiratory and gastrointestinal tracts | Ventilation, the direction of airflow, mechanical filters, electrostatic precipitators, non-thermal plasma air purifiers, photocatalytic oxidation system, UV disinfection, and surface biocides, anti-adhesive, antimicrobial light, touch-free solutions | Dai et al. 2017 |
